# Occludin and collagen IV degradation mediated by the T9SS effector SspA contributes to blood–brain barrier damage in ducks during *Riemerella anatipestifer* infection

**DOI:** 10.1186/s13567-024-01304-y

**Published:** 2024-04-09

**Authors:** Zongchao Chen, Min Zhu, Dan Liu, Mengsi Wu, Pengfei Niu, Yang Yu, Chan Ding, Shengqing Yu

**Affiliations:** 1 Jiangsu Agri-Animal Husbandry Vocational College, Veterinary Bio-Pharmaceutical, Jiangsu Key Laboratory for High-Tech Research and Development of Veterinary Biopharmaceuticals, Taizhou, Jiangsu China; 2 Shanghai Veterinary Research Institute, Chinese Academy of Agricultural Sciences (CAAS), Shanghai, China; 3 Yangzhou You-Jia-Chuang Biotechnology Co., Ltd., Yangzhou, China

**Keywords:** *Riemerella anatipestifer*, blood‒brain barrier, T9SS, SspA, occludin, collagen IV

## Abstract

*Riemerella anatipestifer* infection is characterized by meningitis with neurological symptoms in ducklings and has adversely affected the poultry industry. *R. anatipestifer* strains can invade the duck brain to cause meningitis and neurological symptoms, but the underlying mechanism remains unknown. In this study, we showed that obvious clinical symptoms, an increase in blood‒brain barrier (BBB) permeability, and the accumulation of inflammatory cytokines occurred after intravenous infection with the Yb2 strain but not the mutant strain Yb2ΔsspA, indicating that Yb2 infection can lead to cerebrovascular dysfunction and that the type IX secretion system (T9SS) effector SspA plays a critical role in this pathological process. In addition, we showed that Yb2 infection led to rapid degradation of occludin (a tight junction protein) and collagen IV (a basement membrane protein), which contributed to endothelial barrier disruption. The interaction between SspA and occludin was confirmed by coimmunoprecipitation. Furthermore, we found that SspA was the main enzyme mediating occludin and collagen IV degradation. These data indicate that *R. anatipestifer* SspA mediates occludin and collagen IV degradation, which functions in BBB disruption in *R. anatipestifer-*infected ducks. These findings establish the molecular mechanisms by which *R. anatipestifer* targets duckling endothelial cell junctions and provide new perspectives for the treatment and prevention of *R. anatipestifer* infection.

## Introduction

*Riemerella anatipestifer* is a gram-negative bacterium that causes septicemic diseases in ducks, geese, turkeys, and other fowl [1]. Pathology is initiated when *R. anatipestifer* gains access to the bloodstream, multiplies in the blood, and disseminates into various tissues, causing meningitis, a severe form of invasive disease caused by *R. anatipestifer* infection. Despite antimicrobial therapy, *R. anatipestifer* infection remains a major cause of mortality and severe neurological sequelae in ducks [2, 3]. However, blood‒brain barrier (BBB) damage resulting from *R. anatipestifer* infection is a far less well-studied topic.

All extracellular pathogens that can cause meningitis can result in a high and/or sustained level of bacteraemia to allow bacteria to enter the central nervous system (CNS), a process in which bacterial cells and bacterially infected host cells must pass through the BBB [4, 5]. The BBB functions to maintain CNS homeostasis by tightly regulating the influx and efflux of ions, oxygen, and nutrients between the blood and the brain parenchyma, as well as protecting the CNS against toxins, pathogens, inflammation, injury, and disease [6, 7]. The BBB separates the blood and brain parenchyma and is composed mainly of brain microvascular endothelial cells (BMECs), pericytes, astrocytes, junction complexes, and a basement membrane (BM) [8, 9]. BMECs are the most abundant cell type in the BBB and are connected by tight junctions. Tight junction proteins (TJPs), such as occludin, claudin-5, and zonula occludens-1 (ZO-1), occupy almost all the space between BMECs; they form a natural barrier and are responsible for transendothelial electrical resistance, thus preventing the entry of substances into the parenchyma through the paracellular route [10, 11]. The BM also contributes substantially to vascular barrier function and consists of four main extracellular matrix (ECM) proteins: collagen IV, laminin, nidogen, and perlecan. These ECM proteins are synthesized predominantly by BMECs, pericytes, and astrocytes [12–14].

The type IX secretion system (T9SS) is a complex translocon found in many species of the phylum Bacteroidetes and is associated with bacterial gliding motility, protein secretion, and virulence [15–18]. Bacteria use the T9SS to secrete many cargo proteins, such as SspA, MPPE, and AS87_RS02955, which have been confirmed to be virulence factors [19–22]. SspA was identified as a serine protease with functions in bacterial protease activity and virulence. In this study, we verified that SspA can mediate BBB disruption and promote the occurrence of bacterial meningitis.

## Materials and methods

### Experimental design

All animal care and experimental procedures were approved by the Institutional Animal Care and Use Committee of Shanghai Veterinary Research Institute, the Chinese Academy of Agricultural Sciences (approval no. SV-20220923-Y02), and performed strictly according to the recommendations outlined in the Guide for the Care and Use of Laboratory Animals. One-day-old Cherry Valley ducks were purchased from Zhuang Hang Duck Farm (Shanghai, China), housed in separate cages with free access to food and water, and maintained on a 12 h light/dark cycle at 21 °C (room temperature).

### Bacterial strains

Wild-type *R. anatipestifer* Yb2 is a serotype 2 virulent strain [23]. The *sspA* gene mutant strain Yb2ΔsspA and the complementation strain cYb2ΔsspA were constructed in our previous study [21]. The *R. anatipestifer* strains were grown at 37 °C in tryptic soy broth (TSB; Difco, Franklin Lakes, NJ, USA) or on solid tryptic soy agar (TSA) plates, and TSB was supplemented with 25% glycerol for storage at −80 °C. *Escherichia coli* strains were incubated at 37 °C in Luria broth (LB; Oxoid Ltd). When required, ampicillin (100 μg/mL), erythromycin (1.0 μg/mL), kanamycin (50 μg/mL), ampicillin (100 μg/mL), and cefoxitin (5 μg/mL) were added to the media.

### Cell culture, plasmid construction, and transfection

Mouse brain endothelial bEnd.3 cells (ATCC, Manassas, VA, USA) and human embryonic kidney (HEK) 293 T cells (ATCC) were cultured in Dulbecco’s modified Eagle’s medium (HyClone; GE Healthcare, Little Chalfont, UK) supplemented with 10% foetal bovine serum (Gibco; Thermo Fisher Scientific, Waltham, MA, USA), 100 units/mL penicillin, and 100 μg/mL streptomycin (Invitrogen, Carlsbad, CA, USA) at 37 °C in a humidified atmosphere with 5% CO_2_.

*Occludin* cDNA was amplified from duck brain mRNA, and the *sspA* gene was amplified from Yb2 genomic DNA using high-fidelity Taq polymerase (Vazyme, Nanjing, China). The primers used for *occludin* and *sspA* amplification are listed in Table 1. The *occludin* fragment was inserted into the pcDNA 4.0 expression vector with a C-terminal *myc* tag, and the *sspA* fragment was inserted into the pcMV expression vector with a C-terminal *flag* tag.

**Table 1:** **Primers used for gene amplification**

Gene product (source)	Sequences	Product length (bp)
ZO-1 (duck)	F: 5ʹ-CCAGTAGCTCGACAGGAACC-3′	146
	R: 5ʹ-CTTGCGCTTCGGTCTCTTAC-3′	
Occludin (duck)	F: 5′-AATTCGACACCGACCTCAAG-3′	254
	R: 5′-CTTGTCGTAGTCGCTCACCA-3′	
Laminin-α4 (duck)	F:5′-TGCACACAACCTACGAGGAC-3′	188
	R: 5′-CAGCAGCATCATTGACACGG-3′	
IL-β1 (duck)	F:5′-TCATCTTCTACCGCCTGGAC-3′	244
	R: 5′-GTAGGTGGCGATGTTGACCT-3′	
IL-6 (duck)	F:5′-TTCGACGAGGAGAAATGCTT-3′	150
	R: 5′-CCTTATCGTCGTTGCCAGAT-3′	
IL-8 (duck)	F:5′-TGGTAAGGATGGGAAACGAG-3′	211
	R: 5′-TTGGCCAGAATTGCCTTTAC-3′	
IL-10 (duck)	F:5′-CTGACCTCCTACCAGCGAAG-3′	179
	R: 5′-CTCCATGTAGAACCGCATCA-3′	
IL-17A (duck)	F:5′-TGCCTACGGGAAGGTGATAC-3′	211
	R: 5′-GTGGTCCTCATCGATCCTGT-3′	
IL-17RA (duck)	F:5′-CAAGTCCGCATCTCTCACAA-3′	171
	R: 5′-ATTTGGTACCGCGTTGACTC-3′	
CCL3 (duck)	F:5′-CTTTGCTACGAGAACCTGGC-3′	249
	R: 5′-CTAGCTGGGGATGGAGAAGG-3′	
CCL4 (duck)	F:5′-GCTCTTCTCCTCATTGCAGC-3′	212
	R: 5′-CAGGTGTCACTTGGATTGGC-3′	
CCL5 (duck)	F:5′-CTGTGGCCCATCTTGACAAC-3′	202
	R: 5′-TCAAAGTGCTTCAGGTGTGC-3′	
CCR5 (duck)	F:5′-TGGGCCTACTATGCTGTTCA-3′	230
	R: 5′-GGAACAGAGGCAAGCATAGC-3′	
CX3CL1 (duck)	F:5′-AGCAGCAGTTCCAGAAAAGC-3′	162
	R: 5′-TTGCTGGTCACCTCTGTCAT-3′	
TNFSF13β (duck)	F:5′-TGATGACGAGAAAGCAGGTG-3′	189
	R: 5′-AAGCAGGCCTGTAGCACTGT-3′	
ITGβ2 (duck)	F:5′-AATTCAGGCTACACGGGGAA-3′	175
	R: 5′-CGTAGATCTGCTTGTTGGGC-3′	
PECAM-1 (duck)	F:5′-GTGGGACTGCTTTTCTCTGC-3′	212
	R: 5′-TGGATGGCTTTTCACATTCA-3′	
VCAM-1 (duck)	F:5′-TTCCTTCTGATCGCCTGGAA-3′	239
	R: 5′-TGGGCCAAAGTTTGCATTCA-3′	
SELE (duck)	F:5′-CCGCAACCATAACGCTAAGT-3′	206
	R: 5′-CCTCATTTCCATTGCCATTT-3′	
SELP (duck)	F:5′-CTGCCGCCTTCTTAAAGCAA-3′	177
	R: 5′-TGGTGAAGATGCCTGGTGAT-3′	
β-Actin (duck)	F:5′-GAGAAATTGTGCGTGACATCA-3′	153
	R: 5′-CCTGAACCTCTCATTGCCA-3′	
Occludin (duck)	F:5′-TAGTCCAGTGTGGTGGAATTATGTTCAGCAAGAAGTCCTA-3′	1524
	R: 5′-GCTGGATATCTGCAGAATTCACCCCGCACCTTGTCGTAG-3′	
sspA (*R. anatipestifer*)	F:5′-TGAACCGTCAGAATTAAGCTTATGAAGAAGATCATCTTTGT-3′	2130
	R: 5′-GAATTCGCGGCCGCAAGCTTCTTCTTGATGAACTTCTTAG-3′	
Claudin-5 (bEnd.3)	F: 5′-CGCTTGTGGCACTCTTTGT-3′	168
	R: 5′-ACTCCCGGACTACGATGTTG-3′	
JAM2 (bEnd.3)	F: 5′-CTAGTGGCTCCTGCTGTTCC-3′	108
	R: 5′-GTACTCCGGAGCTGGGTTTC-3′	
VCAM-1 (bEnd.3)	F: 5′-ATTTTCTGGGGCAGGAAGTT-3′	179
	R:5′-ACGTCAGAACAACCGAATCC-3′	
SELE (bEnd.3)	F: 5′-ACACATCTGGTGGCAATTCA-3′	211
	R:5′-TGTTGTTTGGTTCACCTGGA-3′	
β-Actin (bEnd.3)	F: 5′-AGCCATGTACGTAGCCATCC-3′	228
	R:5′-CTCTCAGCTGTGGTGGTGAA-3′	

Plasmids containing the sequence of *occludin* or *sspA* were purified with a midiprep kit (Tiangen, Beijing, China) and transiently transfected into 293 T cells using Lipofectamine 3000 Transfection Reagent (Thermo Fisher Scientific) according to the manufacturer’s instructions. After 36 h of transfection, the cells were lysed in RIPA lysis buffer (Thermo Scientific) containing a protease inhibitor cocktail (Beyotime, Shanghai, China) for 1 h on ice, and the samples were frozen in a −80 °C freezer for protein analysis.

### Infection of animals

Eighty 14-day-old Cherry Valley ducks were randomly divided into four experimental groups: a group infected with Yb2 (Group 1), a group infected with Yb2ΔsspA (Group 2), a group infected with cYb2ΔsspA (Group 3), and an uninfected control group (Group 4). The animals in infection Groups (1–3) were injected with 2.5 × 10^8^ CFU/0.2 mL (2000 LD_50_ CFU of the Yb2 strain) [23] in sterile phosphate-buffered saline (PBS) of Yb2, Yb2ΔsspA, or cYb2ΔsspA through the left jugular vein. The animals in the uninfected control group (4) were injected with sterile PBS via the same route. The time at intravenous injection was defined as zero hours. Then, three of the ducks in Groups 1–4 were sacrificed at 24 and 48 h post-injection (the signs observed in Yb2-infected ducks were listlessness, mild coughing and sneezing, greenish diarrhoea, ataxia, torticollis, head and neck tremors, and death; the main macroscopic change observed was a fibrinous exudate on the heart, liver and air sacs in Yb2-infected ducks); brain tissue samples were collected and divided into four parts, which were stored for enumeration of bacterial colony-forming units (CFU), frozen in liquid nitrogen for gene and protein verification, fixed with 4% paraformaldehyde for haematoxylin–eosin (H&E) staining, or fixed with 2.5% glutaraldehyde for transmission electron microscopy. For the BBB permeability assay, three ducks from each group were randomly selected and injected with 2% Evans blue (EB) solution (Sigma‒Aldrich, USA) via the left jugular vein (150 μL per duck), and these ducks were sacrificed 2 hours post-injection (hpi). The concentration of EB in the brain was measured by the optical density method [24] (Table 2). EB formamide solutions with mass concentrations of 8.0, 4.0, 2.0, 1.0, 0.5, and 0.25 mg/litre were prepared. After incubation at 37 °C for 48 h, the optical density at 630 nm (OD_630_) of each formamide solution was measured with an iMark microplate absorbance reader (Bio-Rad Laboratories, Hercules, CA, USA). The EB concentration and optical density were determined by linear regression, and a standard curve was drawn. The brain tissues were weighed, cut into slices, and incubated in formamide solution at 37 °C for 48 h. The solutions were then centrifuged at 3000 rpm for 15 min. The OD_630_ of each supernatant was determined using multimode microplate readers. Formamide solution was used as a blank control. The concentration of EB in brain tissue was calculated according to the standard curve: EB content (mg/g) = [EB content in brain tissues (mg/litre) × formamide volume (mL)]/[duck brain weight (g)].

### Histopathological analyses

Brain tissues fixed with 4% paraformaldehyde were embedded in paraffin, sectioned at a thickness of 9 μm, and affixed to glass slides. The slides were stained with haematoxylin–eosin, and images were acquired using a Zeiss upright microscope with an attached AxioCam Icc3 camera. Adobe Photoshop and Illustrator software were used to process the images.

### Electron microscopy

Brain tissues were fixed with 2.5% glutaraldehyde, rinsed with PBS, and postfixed in 2% osmium tetroxide at 4 °C for 2 h. After rinsing with water and dehydration through a series of increasing concentrations of ethanol, samples were prepared by embedding in epoxy resin and staining with toluidine blue. Ultrathin Sects. (50 nm) were double stained with uranium acetate and lead citrate and were then observed and photographed with a transmission electron microscope.

### Cell infection

bEnd.3 cells were grown to 90% confluence in 12-well plates and then infected with the *R. anatipestifer* strains Yb2, Yb2ΔsspA, or cYb2ΔsspA at a multiplicity of infection (MOI) of 100, as previously described [25, 26]. After infection with *R. anatipestifer*, a portion of the cells was rinsed twice with PBS (pH 7.4) and lysed with RIPA lysis buffer containing a protease inhibitor cocktail for protein extraction. The other portion was lysed with TRIzol reagent (Invitrogen) for RNA extraction.

The abilities of *R. anatipestifer* to adhere to and invade bEnd.3 cells were determined as previously described [17, 26, 27]. The *R. anatipestifer* strains Yb2, Yb2ΔsspA, and cYb2ΔsspA were incubated in TSB with shaking at 37 °C to OD_600_ ≈ 0.4–0.6. These bEnd.3 cells were then cultured in 12-well cell culture plates and infected with the appropriate strain at an MOI of 100. After 2 h of infection, the abundance of adhered bacteria was quantified by lysing the cells with 0.1% trypsin and spreading the cell lysate onto TSA plates. For the invasion assay, after being infected for 2 h, the cells were washed three times with PBS and incubated for an additional 1 h with DMEM supplemented with 200 µg/mL gentamicin to kill any extracellular bacteria. The number of intracellular bacteria was determined by lysing the cells and counting the bacteria in the cell lysate spread onto TSA plates. The experiments were performed three times with three independent wells per strain.

### Determination of in vitro permeability

A transendothelial permeability assay was carried out with a Transwell system as previously described [26, 28] with modifications. bEnd.3 cells were cultured on 3.0-μm-pore-size Transwell filters (Corning Costar, Cambridge, MA, USA) to 100% confluence. A total of 10^6^ bacterial cells were added to the upper chamber, and the Transwell plate was incubated at 37 °C. Then, 0, 0.25, 0.5, 1.0, and 2 h post-infection, the supernatants in the lower chambers were collected and plated onto solid medium to determine the bacterial counts (CFU). Three measurements of CFU were made at each time point for each sample.

### Total RNA extraction and quantitative RT‒PCR

Total RNA from duck brains and bEnd.3 cells was isolated using TRIzol reagent. The concentrations of RNA were measured with a NanoDrop 1000 spectrophotometer (Thermo Fisher Scientific). cDNA was synthesized from RNA by using the RT Master Mix for qPCR II (gDNA Digester Plus) Reagent Kit (Vazyme, Nanjing, China) according to the manufacturer’s instructions. The cDNA was used for PCR and quantitative PCR (qPCR). The primers designed for qPCR analysis of duck brains and bEnd.3 cells are listed in Table 1. AceQ Universal SYBR qPCR Master Mix (Vazyme) was used for amplifying cDNA. The amplification reactions were prepared as follows: 10.0 μL of SYBR Green Master Mix, 0.5 mL each of the forward and reverse primers, 7.0 μL of ddH_2_O, and 2.0 μL of cDNA. The PCR conditions were 95 °C for 30 s followed by 40 cycles at 95 °C for 15 s and 60 °C for 30 s, 95 °C for 15 s, 60 °C for 1 min, and 95 °C for 15 s. The primer sequences are listed in Table 1. β-Actin was used as an internal reference. The 2^−ΔΔCT^ method was used to calculate relative gene expression levels [29].

### Western blot analysis

Protein expression was measured in duck brain and bEnd.3 cell lysates as previously described [5]. Whole brain tissues and cells from each group were lysed in RIPA buffer and centrifuged at 12 000 × *g* for 10 min at 4 °C. The protein concentration was determined using a Pierce BCA Protein Assay Kit (Thermo Fisher Scientific). Equal amounts of protein (30 μg/lane) were separated by using Tris–HCl sodium dodecyl sulfate‒polyacrylamide gel electrophoresis (SDS‒PAGE) and then transferred onto PVDF membranes (Merck Millipore, Darmstadt, Germany). The membranes were blocked with 5% skim milk in Tris-buffered saline supplemented with 0.1% Tween 20 (TBS-T; Bio-Rad, Hercules, CA, USA) and then incubated with primary antibodies. Horseradish peroxidase-conjugated goat anti-mouse IgG (H^+^L) (1:5000, Abcam, ab6728) and goat anti-rabbit IgG (H^+^L) (1:5000, Abcam, ab6721) were used as secondary antibodies. Following incubation, the bound antibodies were visualized using High sig ECL Western blot substrate (Tanon, China). Immunoreactive band densities were quantified using Quantity One software (Bio-Rad Laboratories, USA). Protein expression was then quantified using Quantity One software (Bio-Rad Laboratories). The primary antibodies (anti-Occludin: 1:1000, Proteintech, 27260-1-AP; anti-ZO-1: 1:1000, Proteintech, 21773-1-AP; anti-VCAM-1: 1:1000, Beyotime, AF1021; anti-SELE: 1:1000, Beyotime, AF7959; anti-COL4A1: 1:1000, Signalway Antibody, 40773; anti-β-actin: 1:10 000, Proteintech, 66009-1-Ig) used in this study were diluted in PBS containing 3% BSA and 0.1% Tween 20 (Sigma) and incubated overnight at 4 °C with the membranes (Table 2).

**Table 2:** **Samples used for different experiments**

Time point	Sample characteristics			Experiments
24 h	28	Divided into four parts	28	Bacterial enumeration
			28	Gene and protein verification
			12	Haematoxylin–eosin staining
			0	TEM observation
	12	Divided into two parts	12	Bacterial enumeration
			12	BBB permeability assay
48 h	28	Divided into four parts	28	Bacterial enumeration
			28	Gene and protein verification
			12	Haematoxylin–eosin staining
			12	TEM observation
	12	Divided into two parts	12	Bacterial enumeration
			12	BBB permeability assay

### Immunofluorescence experiments

293 T cells were grown on 12-well coverslips and then transiently co-transfected with the plasmids pcDNA-*occludin-myc* and pcMV-*sspA-flag*. Cells on coverslips were fixed with 4% paraformaldehyde for 30 min, washed three times with PBS, permeabilized with 0.2% Triton X-100 for 10 min, and washed again three times with PBS. The coverslips were then incubated with a rabbit anti-MYC-C tag polyclonal antibody (1:200, Proteintech, 16286–1-AP) and a mouse anti-FLAG-C tag monoclonal antibody (1:200, Proteintech, 66008–4-Ig) diluted in PBS (5% BSA) for 2 h, washed three times with PBS, incubated with Goat Anti-Rabbit IgG H&L (Alexa Fluor 488) (Abcam, ab150077) and Goat Anti-Mouse IgG H&L (Alexa Fluor 647) (Abcam, ab150115) (both 1:1000 diluted in PBS containing 5% BSA) as the secondary antibody for 1 h, and washed with PBS. The coverslips were then incubated with 0.1 μg/mL DAPI diluted in PBS for 10 min and rinsed with PBS. The coverslips were then mounted and observed with a fluorescence microscope (Olympus DP73, Japan).

### Immunoprecipitation

Cells co-transfected with pcMV*-sspA-flag* and pcDNA*-occludin-myc* were washed with ice-cold PBS and lysed with RIPA buffer supplemented with protease inhibitor cocktail. The cell lysates were centrifuged at 12 000 rpm for 20 min. Next, the supernatants were incubated with 50 μL of Anti-Flag Magnetic Beads (MedChemExpress, USA) at 4 °C overnight. The beads were rinsed five times with TBS-T, and then 50 μL of protein loading buffer was added. The immunoprecipitated proteins were subsequently analysed by Western blot using a rabbit anti-MYC-C tag polyclonal antibody.

### Gelatine zymography

After transfection of pcMV*-sspA-flag* into 293 T cells, the cell lysate was analysed by gelatine zymography as we described previously [21, 30]. In brief, equal amounts of (50 μg) of protein in cell lysates were separated via SDS‒PAGE at 4 °C on a 10% polyacrylamide gel copolymerized with gelatine (1%) as the substrate. The gel was washed with elution buffer (containing 2.5% Triton X-100, 5 mmol/L CaCl_2_, and 1 μmol/L ZnCl_2_ solution) and washing buffer (containing 5 mmol/L CaCl_2_ and 1 μmol/L ZnCl_2_ solution) for 20 min each. The gel was then incubated for 18–20 h at 37 °C in incubation buffer (50 mmol/L Tris–HCl buffer [pH 7.4] containing 5 mmol/L CaCl_2_, 1 μmol/L ZnCl_2_, and 0.02% Brij-35 solution). The gel was stained with 0.05% Coomassie Brilliant Blue R-250 and then destained with 30% methanol and 10% acetic acid. Gelatinolytic activity was detected as unstained bands against the background of Coomassie-stained gelatine, and the recombinant protein rSspA, which was confirmed to exhibit hydrolytic activity towards gelatine, was used as the gelatinase control [21]. The lysates of untransfected 293 T cells and 293 T cells transfected with the pcMV*-flag* plasmid were used as negative controls.

### Hydrolytic activity assays

293 T cells were cultured in 6-well cell culture plates and co-transfected with pcDNA*-occludin-myc* (2.5 μg/well) and pcMV*-sspA-flag* (0, 0.5, 1.0, 1.5, 2.0, or 2.5 μg/well). After 36 h of co-transfection, the cells were lysed with RIPA lysis buffer and collected for Western blot analysis.

The secreted proteins of the wild-type strain Yb2, mutant strain Yb2ΔsspA, and complementation strain cYb2ΔsspA were collected as previously described [17]. In brief, the strains were incubated in ADCF-mAb medium (HyClone) for 8 h at 37 °C with shaking at 220 rpm, after which 5 mL samples of medium were collected and centrifuged at 4 °C and 19 950 × *g* for 10 min. The resulting cell-free supernatants were subsequently purified by passage through 0.22 µM HT Tuffryn® syringe filters (Pall Life Sciences, Ann Arbor, MI, USA).

Occludin-MYC, which was harvested from pcDNA*-occludin-myc*-transfected 293 T cells, and collagen IV (Sigma, 9007-34-5) were incubated with proteins secreted by the wild-type strain Yb2, mutant strain Yb2ΔsspA, or complementation strain cYb2ΔsspA or with recombinant SspA at an enzyme:substrate weight ratio of 1:1000 at 37 °C in buffer (100 mM Tris, 150 mM NaCl, 5 mM CaCl_2_, 0.02% NaN_3_; pH 8.0). At specific time points, aliquots were collected for Western blot analysis or SDS‒PAGE.

### Statistical analysis

All experimental values are expressed as the means and standard errors of the mean (SEMs). In all analyses, a *p* value < 0.05 was considered to indicate statistical significance. Statistical significance was determined using one-way ANOVA for comparisons among multiple groups or the *t* test for comparisons between two groups (GraphPad Prism 7).

## Results

### *R. anatipestifer-*induced BBB disruption and encephalitis are correlated with T9SS-mediated secretion of SspA

**Figure 1:**
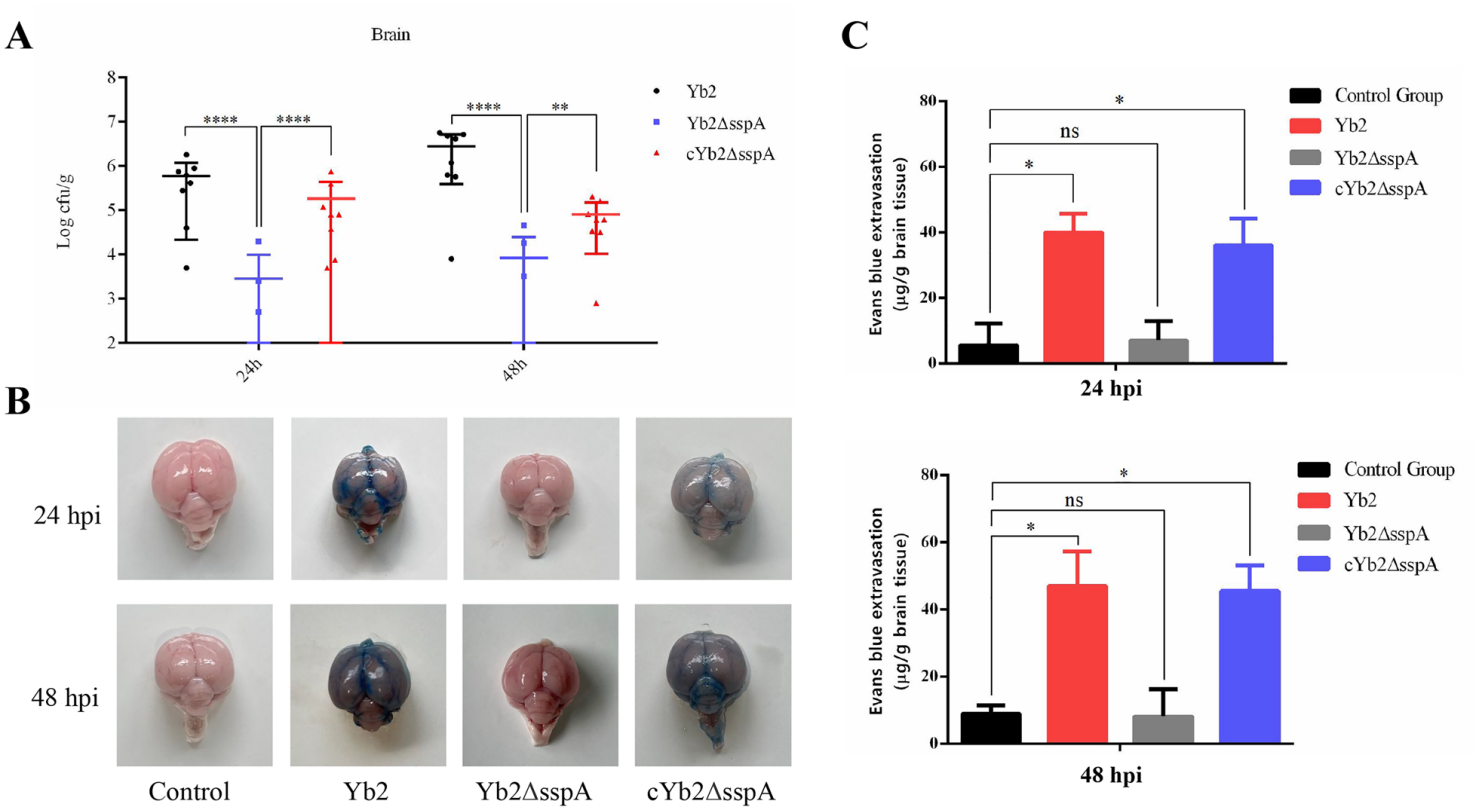
**Bacterial loads in the brain and BBB permeability in ducks post *R. anatipestifer* infection.** **A** The bacterial load in the brain tissues of ducks infected with Yb2, Yb2ΔsspA, and cYb2ΔsspA was measured 24 and 48 h post-infection. The data are presented as the means ± standard deviations of ten animals from triplicate experiments. Statistical significance was assessed using two-tailed Student’s *t* test. The asterisks indicate the level of statistical significance (**, *p* < 0.01; ****, *p* < 0.0001). **B** Three ducks each from Groups 1–4 were injected intravenously with 2% Evans blue solution (0.15 mL) and sacrificed 2 h post-injection. Representative images are shown in the figure. Blue staining in the brain indicates that the BBB was destroyed during infection. **C** The amounts of Evans blue dye in the brains of ducklings infected with different strains. The data are presented as the means ± standard deviations of three animals from triplicate experiments. Statistical significance was assessed using one-way analysis of variance (ANOVA). The asterisks indicate the level of statistical significance (*, *p* < 0.05). ns: not significant.

To determine whether SspA contributes to BBB disruption and encephalitis in vivo, we established a duck model of *R. anatipestifer* infection [31] in which 14-day-old Cherry Valley ducks were intravenously injected with 2.5 × 10^8^ CFU of Yb2, Yb2ΔsspA, or cYb2ΔsspA per duck. At the experimental endpoint, the ducks were euthanized, and brain tissue was collected to determine the bacterial load. We observed similar numbers of recovered Yb2 and cYb2ΔsspA cells but a significant decrease in the number of recovered Yb2ΔsspA mutant cells in brain tissue (Figure 1A).

BBB integrity was monitored by perfusion with Evans blue (EB) dye solution. There was noticeable EB staining in the brains of ducks infected with Yb2 and cYb2ΔsspA at 24 and 48 hpi, and the EB content was significantly greater in these ducks than in the uninfected and Yb2ΔsspA-infected ducks (Figure 1B). The EB content in the brain of each group was calculated and is shown in Figure 1C.

**Figure 2:**
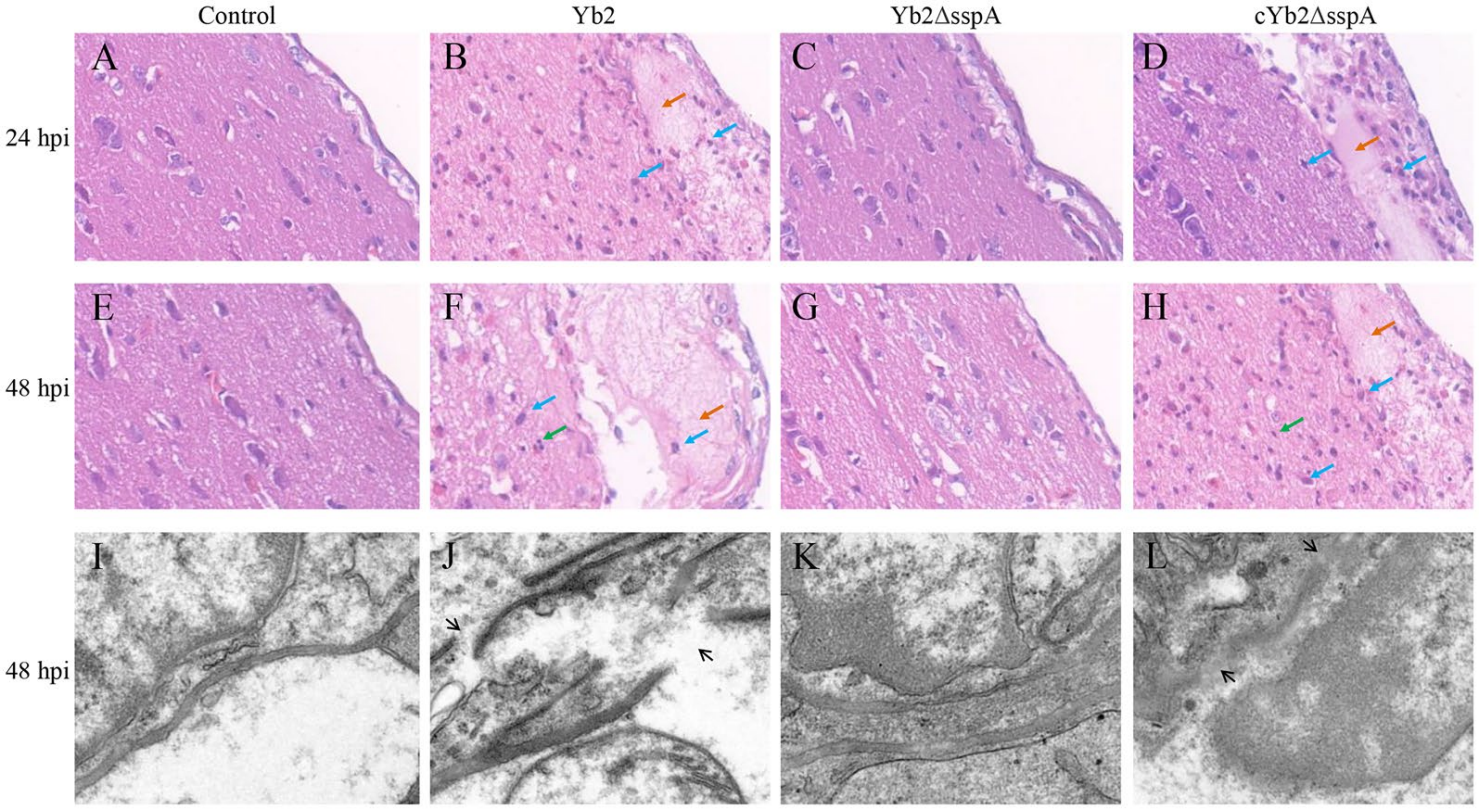
**Pathological evaluation of the duck brain and BBB after *R. anatipestifer* infection.** Fourteen-day-old Cherry Valley ducks were intravenously injected with 2.5 × 10^8^ CFU of *R. anatipestifer* Yb2, Yb2ΔsspA, or cYb2ΔsspA. Histopathological changes in the brain and changes in the BBB ultrastructure were examined by H&E staining and transmission electron microscopy, respectively, at the indicated time points after infection. **A**–**H** Brain samples from control ducks and Yb2-, Yb2ΔsspA-, or cYb2ΔsspA-infected ducks were stained with H&E 24 h (**A**–**D**) and 48 h (**E** to **H**) post-infection. Blue arrow, inflammatory cell infiltration; orange arrow, meningeal thickening; green arrow, microglial proliferation. **I**–**L** The ultrastructure of the impaired duckling BBB observed by transmission electron microscopy. **I** Normal duck brain control; **J** duck brain with Yb2 infection. The intercellular junctions (TJs) were altered, and the basement membrane (black arrow) was disrupted. **K** The brain of a Yb2ΔsspA-infected duck was similar to that of a normal control duck brain; the TJs were integrated, and the basement membrane was intact. **L** Duck brain with cYb2ΔsspA infection. The TJs were altered, and the basement membrane (black arrow) was disrupted.

Histopathological examination of fixed tissue revealed meningeal thickening as well as the presence of inflammatory infiltrates in the brains of ducks infected with Yb2 and cYb2ΔsspA at 24 hpi (Figures 2B, D) and 48 hpi (Figures 2F–H). No differences in clinical symptoms and histopathological features were found between the control group and Yb2ΔsspA group ducks at 24 hpi (Figures 2A–C) or 48 hpi (Figures 2E and G).

The BBB integrity in the ducks was explored using transmission electron microscopy (TEM). The results showed that the tight junctions in the BBB were impaired and that the basement membrane was ruptured in Yb2- and cYb2ΔsspA-infected ducks (Figures 2J and L). However, no obvious BBB destruction was detected in the control ducks or Yb2ΔsspA-infected ducks (Figures 2I and K). These data indicate that the destruction of the BBB occurred during *R. anatipestifer* infection and that the entry of bacteria into the CNS may be related to the direct effects of SspA on BMECs.

### SspA changed the expression of TJPs, BM proteins and CAM in vivo

**Figure 3:**
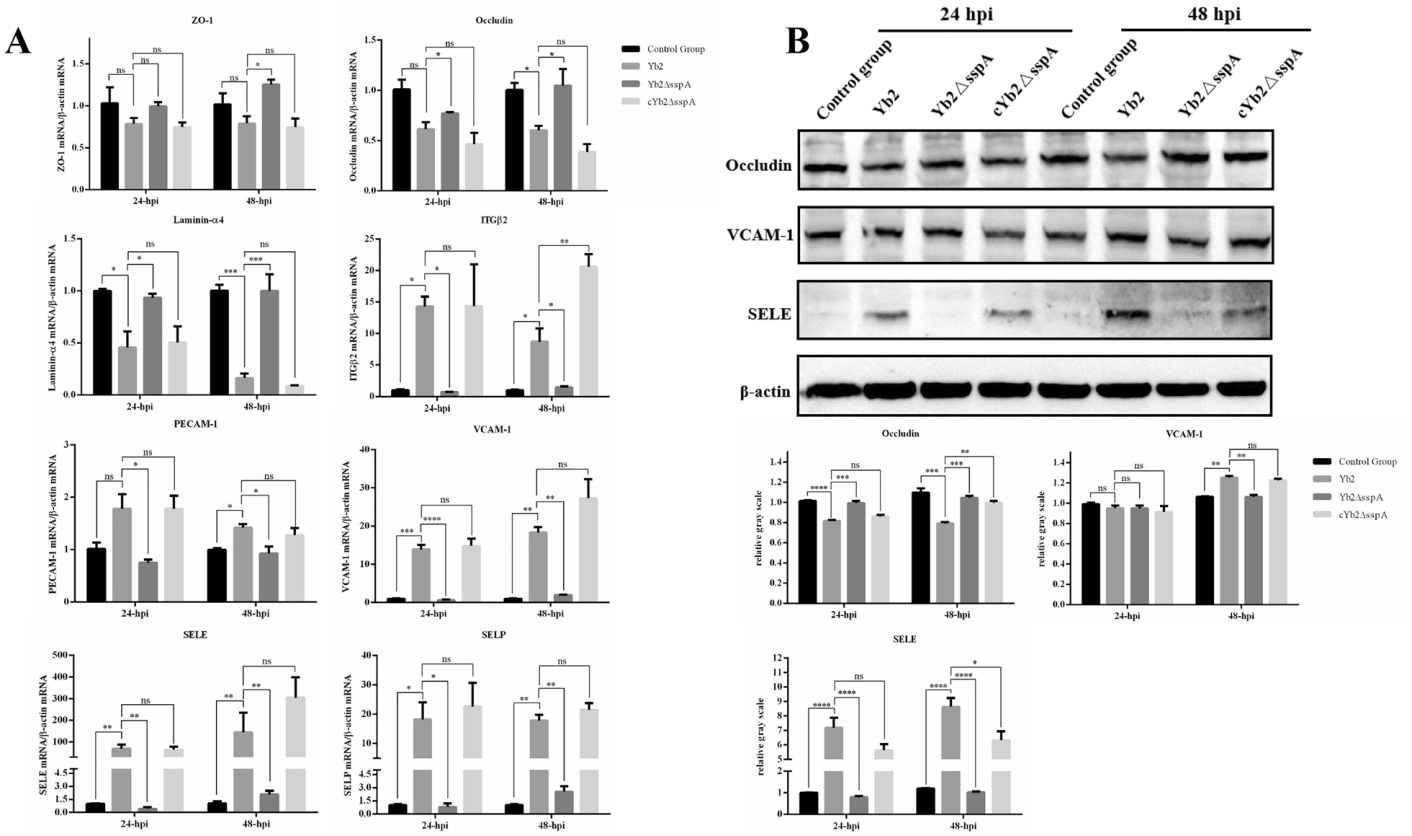
**SspA affects the expression of TJPs, BM proteins and CAMs in the brain.** **A** Changes in the expression of these molecules at the transcriptional level. mRNA expression was measured by qPCR, and relative expression was calculated using the 2^−ΔΔCt^ method. Compared with those in animals infected with the wild-type strain Yb2, the mRNA levels of ITG-β2, PECAM-1, VCAM-1, SELE, and SELP were significantly lower and the levels of ZO-1, occludin, and laminin-α were significantly greater in animals infected with the SspA deletion mutant strain Yb2ΔsspA. **B** Western blot analysis of protein expression using the Quantity One program. Duck *β*-actin was used as a normalization control in the analyses. The data are presented as the means ± standard deviations of three animals from triplicate experiments. Statistical significance was assessed using one-way analysis of variance (ANOVA). The asterisks indicate the level of statistical significance (*, *p* < 0.05; **, *p* < 0.01; ***, *p* < 0.001; ****, *p* < 0.0001).

The integrity of the BBB is controlled by TJPs, junctional adhesion molecules (JAMs), and BM proteins in BMECs. Therefore, the expression of these proteins and their encoding genes was examined in cells throughout the process of *R. anatipestifer* infection. Expression analysis of selected genes encoding TJPs, JAMs, and BM proteins was performed on duck brains harvested throughout the timeline of *R. anatipestifer* infection (Figure 3A). The mRNA/protein levels of ZO-1, occludin, and laminin-α4 in the Yb2- and cYb2ΔsspA-infected ducks were significantly lower than those in the control ducks and Yb2ΔsspA-infected ducks at 24 and 48 hpi (Figures 3A, B). The findings described above reveal that the TJPs and BM proteins play complex roles in *R. anatipestifer* infection.

Cell adhesion molecules (CAMs) function by targeting leukocytes to allow their adherence to and passage through the BBB. qPCR and Western blot analyses were used to examine and verify the functions of CAMs in *R. anatipestifer* infection. The expression levels of vascular endothelial cell adhesion molecule 1 (VCAM-1) and platelet endothelial cell adhesion molecule 1 (PECAM-1) in the Yb2- and cYb2ΔsspA-infected ducks were significantly greater than those in the control and Yb2ΔsspA-infected ducks at 24 and 48 hpi (Figures 3A, B). The expression of E-selectin (SELE), P-selectin (SELP), and integrin β2 (ITGβ2) also increased gradually in the Yb2- and cYb2ΔsspA-infected ducks as the infection time increased (Figures 3A, B).

### SspA promotes the expression of inflammatory factors in vivo

**Figure 4:**
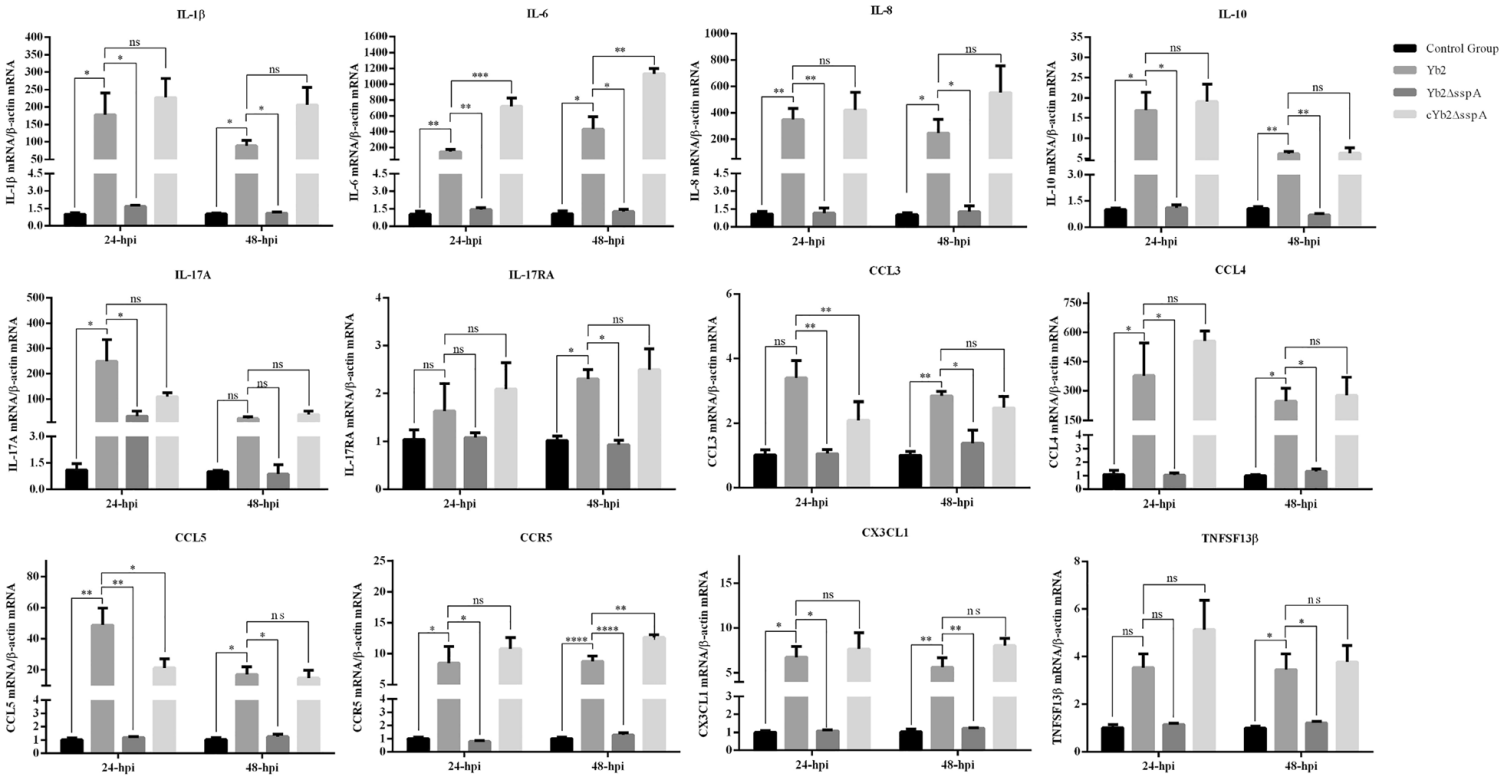
**SspA increases inflammatory factor expression in the brain.** The mRNA expression of inflammatory factors was measured by qPCR, and relative expression was calculated using the 2^−ΔΔCt^ method. Duck β-actin was used as a normalization control. The mRNA levels of IL-1β, IL-6, IL-8, IL-10, IL-17A, IL-17RA, CCL3, CCL4, CCL5, CCR5, CX3CL1, CX3CL1, and CX3CR1 were significantly lower in the animals infected with the SspA deletion mutant strain Yb2ΔsspA than in the animals infected with the wild-type strain Yb2. The data are presented as the means ± standard deviations of three animals from triplicate experiments. Statistical significance was assessed using one-way analysis of variance (ANOVA). The asterisks indicate the level of statistical significance (*, *p* < 0.05; **, *p* < 0.01; ***, *p* < 0.001; ****, *p* < 0.0001).

When encephalitis occurs, the BBB is completely destroyed, and cytokine‒cytokine receptor expression is most likely increased in the brain. The mRNA levels of several cytokines were measured in this study. Overall, their expression levels were increased in the Yb2- and cYb2ΔsspA-infected duck brains (Figure 4). Notably, the levels of chemokines such as C–C motif ligand 3 (CCL3), CCL4, and CCL5 were significantly greater in the brains of Yb2- and cYb2ΔsspA-infected ducks than in the brains of control and Yb2ΔsspA-infected ducks at 24 and 48 hpi (Figure 4). The expression of the receptor for these chemokines, CCR5, was significantly greater in the brains of Yb2- and cYb2ΔsspA-infected ducks than that in the brains of control and Yb2ΔsspA-infected ducks. The expression levels of interleukin 1 beta (IL-1β), IL-6, IL-8, IL-10, IL-17A, and the interleukin receptor IL-17RA were significantly increased after infection with Yb2 and cYb2ΔsspA and were much greater than those in the brains of control and Yb2ΔsspA-infected ducks at 24 and 48 hpi (Figure 4).

These data showed that severe inflammation in the brain occurred after infection with Yb2 and cYb2ΔsspA and was accompanied by severe destruction of the BBB, indicating that there was a relationship between inflammation and BBB damage.

### SspA affects bacterial adherence to and passage through a bEnd.3 cell monolayer in vitro

**Figure 5:**
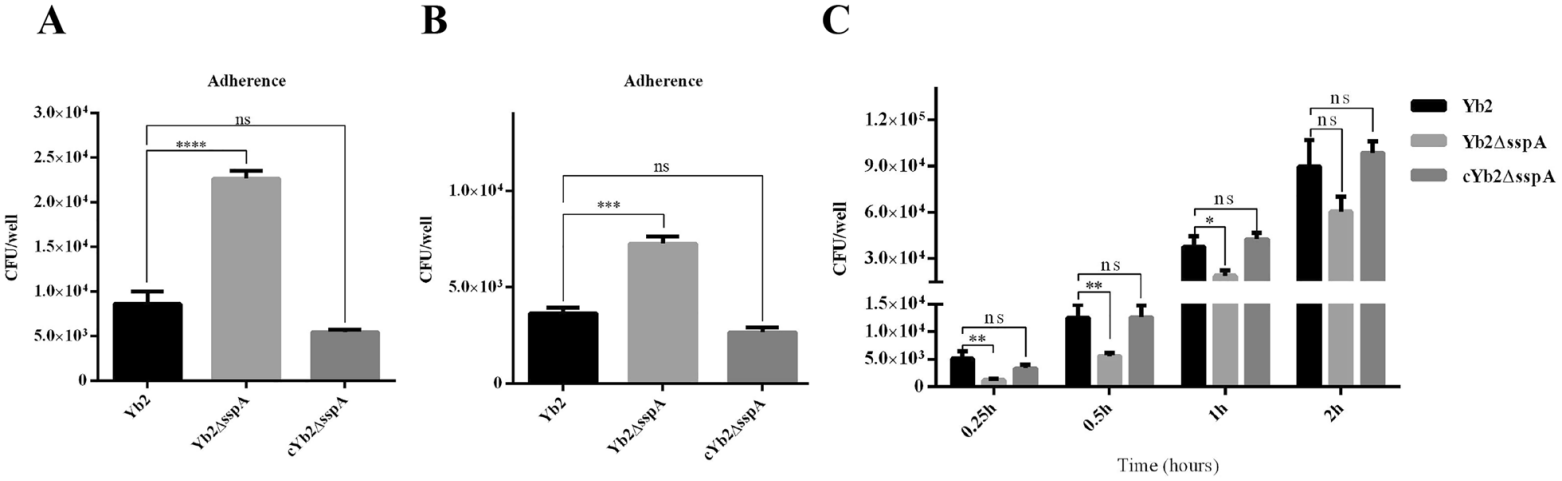
**SspA inhibits bacterial adherence and invasion but enhances bacterial migration across a bEnd.3 cell monolayer*.*** Deletion of *sspA* increased bacterial adhesion to (**A**) and invasion of (**B**) the bEnd.3 cell monolayer but decreased the ability of bacteria to migrate across the cell monolayer in vitro (**C**). The data are presented as the means ± standard deviations of the results of three independent experiments conducted in triplicate. Statistical significance was assessed using one-way analysis of variance (ANOVA). The asterisks indicate the level of statistical significance (*, *p* < 0.05; **, *p* < 0.01; ***, *p* < 0.001; ****, *p* < 0.0001).

The adherence and invasion abilities of the wild-type strain Yb2, mutant strain Yb2ΔsspA, and complementation strain cYb2ΔsspA were evaluated in bEnd.3 cells in vitro. As shown in Figures 5A, B, the adherence and invasion abilities of Yb2ΔsspA were increased compared with those of Yb2. The adherence and invasion abilities of the complementation strain cYb2ΔsspA were restored to the levels of the Yb2 strain.

To determine the ability of *R. anatipestifer* to migrate across the BBB, an in vitro monolayer BBB model that reproduces the main characteristics of the barrier was established using bEnd.3 cells, and the abundance of *R. anatipestifer* released into the lower chamber supernatants (LCSs) was quantified. Bacteria of all strains had crossed the bEnd.3 cell monolayer after 0.25 h of incubation. Thereafter, the number of bacteria crossing the monolayer increased with increasing incubation time. However, Yb2 and cYb2ΔsspA had a much greater ability than Yb2ΔsspA to cross the bEnd.3 cell monolayer (Figure 5C). These results indicated that SspA expression was important for the ability of *R. anatipestifer* to cross the bEnd.3 cell monolayer.

### SspA altered the expression of TJPs, BM proteins, and CAMs in mBMECs in vitro

**Figure 6:**
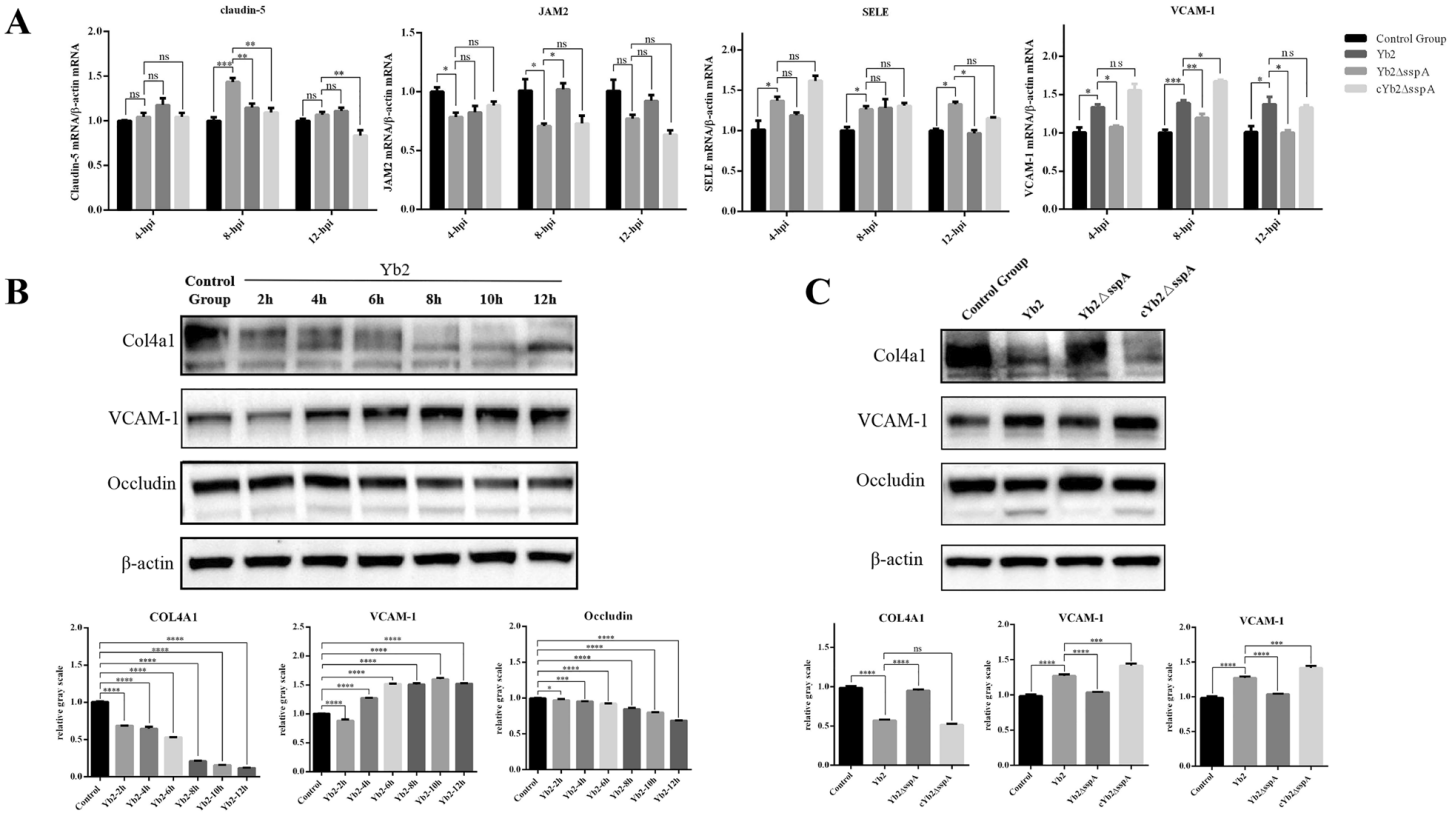
**SspA affects the expression of TJPs, BM, and CAMs in bEnd.3 cells.** **A** qPCR analysis of mRNA expression. mRNA expression was measured by qPCR, and relative mRNA expression was calculated using the 2^−ΔΔCt^ method. Compared with those in cells infected with the wild-type strain Yb2, the mRNA levels of claudin-5, SELE, and VCAM-1 were significantly lower and the level of JAM-2 was significantly greater in cells infected with the SspA deletion mutant strain Yb2ΔsspA. **B** Western blot analysis of protein expression in Yb2‐infected cells at different time points. **C** Comparative analysis of protein levels in Yb2-, Yb2ΔsspA- and cYb2ΔsspA‐infected bEnd.3 cells. The expression of proteins was analysed using the Quantity One program. Mouse *β*-actin was used as a normalization control in both experiments. The data are presented as the means ± standard deviations of three independent experiments conducted in triplicate. Statistical significance was assessed using one-way analysis of variance (ANOVA). The asterisks indicate the level of statistical significance (*, *p* < 0.05; **, *p* < 0.01; ***, *p* < 0.001; ****, *p* < 0.0001).

It has been confirmed that *R. anatipestifer* can infect bEnd.3 cells and cross the bEnd.3 cell monolayer, and its effects on TJPs, BMs, and CAMs were further investigated. As shown in Figure 6A, the expression of TJPs and BMs decreased to various degrees during infection. Moreover, the expression level of VCAM-1 was significantly greater in Yb2- and cYb2ΔsspA-infected cells than in control and Yb2ΔsspA-infected cells. The expression level of SELP in Yb2- and cYb2ΔsspA-infected cells was increased at 12 hpi. The Western blot results showed that the levels of occludin and collagen IV in Yb2- and cYb2ΔsspA-infected cells decreased gradually with increasing infection time compared with those in control and Yb2ΔsspA-infected cells (Figures 6B, C).

### Interaction between SspA and occludin

**Figure 7:**
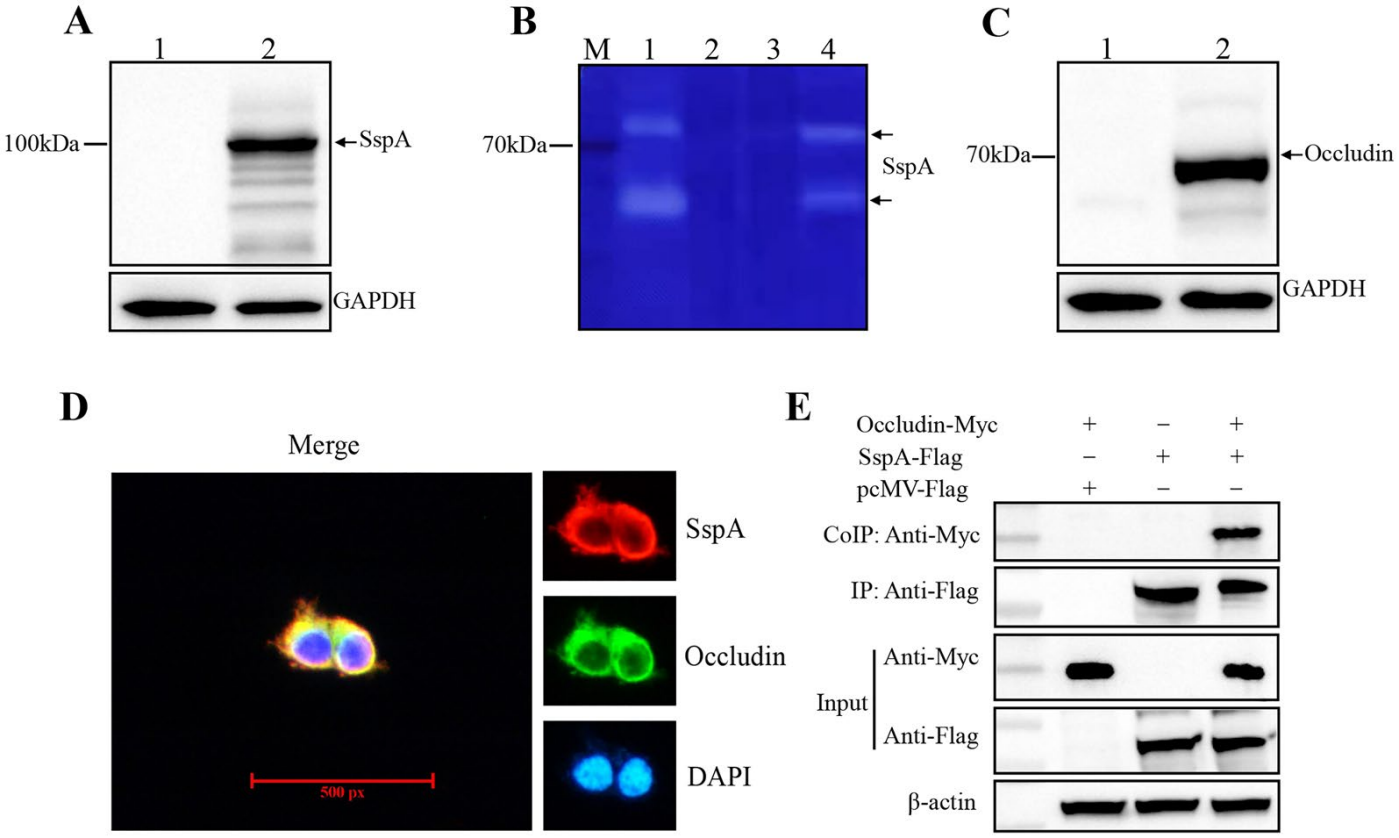
**SspA binds to occludin.** **A** SspA expression in 293 T cells (Lane 1: lysate of 293 T cells transfected with the plasmid pcMV-*flag*; Lane 2: lysate of 293 T cells transfected with the plasmid pcMV-*sspA*-*flag*). **B** SspA has hydrolytic activity towards gelatine (Lane 1: 2 µg of recombinant rSspA protein; Lane 2: lysate of 293 T cells; Lane 3: lysate of 293 T cells transfected with the plasmid pcMV-*flag*; Lane 4: lysate of 293 T cells transfected with the plasmid pcMV-*sspA*-*flag*). **C** Occludin expression in 293 T cells (Lane 1: lysate of 293 T cells transfected with the plasmid pcDNA*-myc*; Lane 2: lysate of 293 T cells transfected with the plasmid pcDNA*-occludin-myc*). **D** The subcellular distribution of SspA (red) and occludin (green) was examined by immunostaining with anti-FLAG-C or anti-MYC-C antibodies. Nuclei were stained with DAPI (blue). Merged images showing SspA/occludin/DAPI fluorescence. **E** Immunoprecipitation of SspA and occludin. 293 T cells were co-transfected with pcMV*-sspA-flag* and pcDNA*-occludin-myc.* The cell lysates were incubated with anti-Flag magnetic beads and subjected to immunoprecipitation using an anti-MYC-C tag polyclonal antibody. The precipitated proteins and whole-cell lysates were analysed by Western blotting.

As SspA decreased the expression of occludin, we next investigated whether SspA directly binds to occludin. 293 T cells were transfected with pcMV*-sspA-flag* or pcDNA*-occludin-myc* and the transfection efficacy was then evaluated via Western blot analysis. The results showed that SspA-FLAG and occludin-MYC were successfully expressed in 293 T cells (Figures 7A, C), and the gelatine zymography assay confirmed that SspA-FLAG exhibited hydrolytic activity towards gelatine (Figure 7B). Next, 293 T cells were co-transfected with pcMV*-sspA-flag* and pcDNA*-occludin-myc*, followed by immunostaining and immunoprecipitation. Immunostaining showed that SspA-FLAG and occludin-MYC were diffused evenly in the cytoplasm of co-transfected 293 T cells. Diffuse cytoplasmic signals of SspA-FLAG and occludin-MYC overlapped (Figure 7D). Immunoprecipitation was carried out by using anti-FLAG magnetic beads (MedChemExpress, NJ, USA). Western blot analysis with an anti-MYC-C antibody showed successful precipitation of occludin (Figure 7E). As expected, no occludin was pulled down with the anti-FLAG tag antibody in mock-co-transfected cells (293 T cells co-transfected with pcMV*-flag* and pcDNA*-occludin-myc*). These results demonstrate the direct binding of these two proteins.

### SspA degrades occludin and collagen IV by hydrolysis

**Figure 8:**
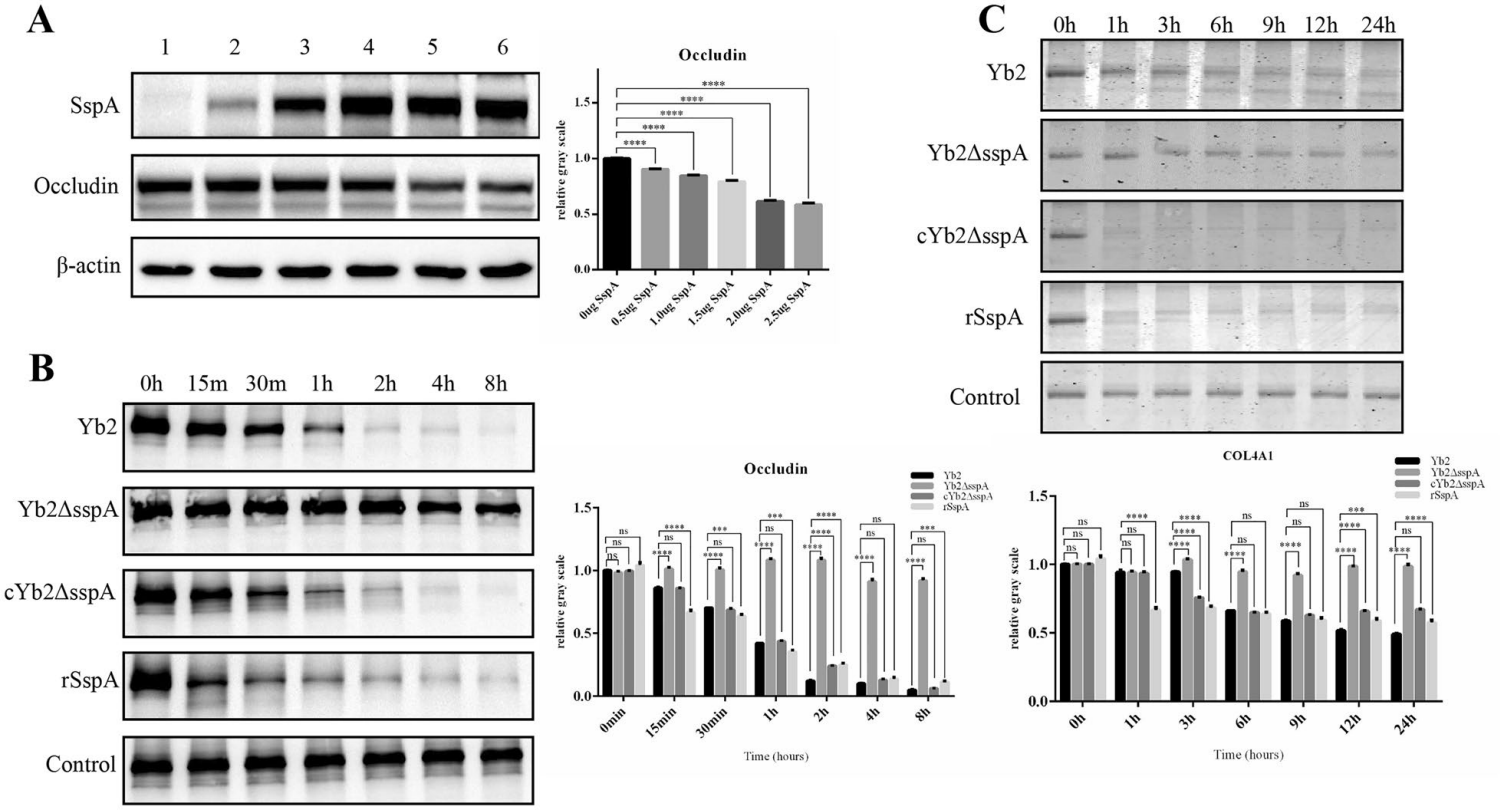
**SspA degrades occludin and collagen IV.** **A** pcDNA*-occludin-myc* (2.5 µg) was co-transfected with pcMV*-sspA-flag* (0, 0.5, 1.0, 1.5, 2.0, or 2.5 µg; corresponding to Lanes 1–6). The occludin level was measured by Western blotting, and data were analysed using the Quantity One program. The results showed that with increasing expression of SspA, the expression of occludin decreased gradually. **B** Occludin was incubated with the secreted proteins of Yb2, Yb2ΔsspA, or cYb2ΔsspA or with recombinant SspA at an enzyme:substrate weight ratio of 1:1000 at 37 °C in buffer (100 mM Tris, 150 mM NaCl, 5 mM CaCl_2_, 0.02% NaN3; pH 8.0). At specific time points, aliquots were collected for Western blotting and data analysis using the Quantity One program. Occludin incubated with ADCF-mAb medium was used as the control. **C** SspA degrades collagen IV. Collagen IV was incubated with the secreted proteins of Yb2, Yb2ΔsspA, or cYb2ΔsspA or with recombinant SspA at an enzyme:substrate weight ratio of 1:1000 at 37 °C in buffer (100 mM Tris, 150 mM NaCl, 5 mM CaCl_2_, 0.02% NaN_3_; pH 8.0). At specific time points, aliquots were collected for Western blotting and data analysis using the Quantity One program. Collagen IV incubated with ADCF-mAb medium was used as the control. The data are presented as the means ± standard deviations of the results of three independent experiments conducted in triplicate. Statistical significance was assessed using one-way or two-way analysis of variance (ANOVA). The asterisks indicate the level of statistical significance (***, *p* < 0.001; ****, *p* < 0.0001). ns: not significant.

293 T cells were co-transfected with pcMV*-sspA-flag* and pcDNA*-occludin-myc.* Briefly, a constant amount (2.5 µg) of pcDNA*-occludin-myc* was co-transfected with gradually increasing quantities (0.5–2.5 µg) of pcMV*-sspA-flag* in each well. Western blot analysis revealed that as the expression of SspA increased, the expression of occludin decreased gradually (Figure 8A).

The ability of SspA to cleave occludin was investigated. Briefly, proteins secreted by the wild-type strain Yb2, mutant strain Yb2ΔsspA, and complementation strain cYb2ΔsspA, as well as the rSspA protein, were incubated with occludin-MYC isolated from 293 T cells transfected with pcDNA-*occludin-myc*, and occludin degradation was evaluated after 0, 15 min, 30 min, 1 h, 2 h, 4 h, and 8 h of incubation. As shown in Figure 8B, the proteases present in the secretome of the Yb2 and cYb2ΔsspA strains, as well as the rSspA protein, were able to degrade occludin. Degradation occurred rapidly, and a marked reduction in the band density was observed after incubation for 15 min. The Yb2ΔsspA mutant strain showed no effective degradation activity before 2 h of incubation, similar to the findings in the control samples (Figure 8B). Therefore, the SspA protease degrades occludin, possibly disrupting TJP integrity and contributing to BBB destruction during *R. anatipestifer* infection.

SspA can downregulate the expression of collagen IV. Collagen IV is the most abundant component of the BM and plays a crucial role in vascular integrity [32]. In this study, collagen IV was used as a substrate to investigate whether *R. anatipestifer* SspA functions in BM degradation. Secreted proteins from the wild-type strain Yb2, mutant strain Yb2ΔsspA, and complementation strain cYb2ΔsspA, as well as the rSspA protein, were incubated with collagen IV, and its degradation was evaluated after 0, 1, 3, 6, 9, 12, and 24 h of incubation. As shown in Figure 8C, the proteases in the secretome of the Yb2 and cYb2ΔsspA strains, as well as the rSspA protein, exhibited activity toward collagen IV, and degradation occurred rapidly after 1 h of incubation. The mutant strain Yb2ΔsspA showed no effective degradation activity before 12 h of incubation, similar to the findings in the control samples. These results indicate that SspA can degrade the BM component collagen IV, which may further disrupt BBB integrity during *R. anatipestifer* infection.

## Discussion

Many bacteria cause nerve damage in the host [33–36]. Bloodborne bacteria that can invade the CNS, such as those causing meningitis, have developed specific mechanisms to circumvent the BBB. Thus, many studies aimed at understanding these mechanisms have been carried out, and thus far, bacteria have been confirmed to cross the BBB by the transcellular route, by the paracellular route, and/or via the Trojan horse mechanism [33, 37–40]. Understanding how *R. anatipestifer* changes BBB permeability in the host and results in barrier dysfunction will aid in the development of new strategies to prevent CNS infection. Although numerous studies have focused on *R. anatipestifer* virulence factors, the pathogenic mechanisms by which *R. anatipestifer* invades the CNS and causes meningitis are unclear.

It has been reported that *R. anatipestifer* can enter the CNS [41]. However, no studies have shown that the BBB is impaired when *R. anatipestifer* enters the CNS. In the present study, we used in vitro and in vivo infection models to elucidate how the BBB is damaged during *R. anatipestifer* infection. Our findings directly demonstrated that *R. anatipestifer* infection can lead to severe impairment of BBB integrity and significantly increase BBB permeability both in vivo and in vitro.

We next investigated whether and how *R. anatipestifer* infection contributes to BBB damage. We found that *R. anatipestifer* infection induced rapid SspA-mediated degradation of the TJ protein occludin and the BM protein collagen IV, which contributed to bacterial infection-induced BBB disruption. TJs are important structural components of the BBB that seal the gaps between adjacent endothelial cells. Certain transmembrane proteins, i.e., claudins and occludin, are key molecules that form these seals [11, 42]. The BM also contributes substantially to BBB integrity, and collagen IV is the most abundant component of the BM [13, 43–46]. SspA is a metalloproteinase secreted via the *R. anatipestifer* T9SS. In our previous study, *R. anatipestifer* SspA was verified to be involved in bacterial virulence and the degradation of host gelatine, fibrinogen, and bacitracin LL-37 [21]. Bacteria-secreted metalloproteases can disrupt host BBB integrity, e.g., *Bacillus anthracis* InhA contributes to BBB disruption associated with anthrax meningitis through proteolytic attack of the endothelial tight junctional protein zonula occludens (ZO)-1 [47]. In addition, host matrix metalloproteinases (MMPs) are reportedly involved in the pathophysiology of bacterial meningitis [48]. In addition to these effects of bacteria, as reported in SARS-CoV-2-infected BMECs, increased expression of MMP9 also mediates the degradation of collagen IV, which promotes the BBB penetration of SARS-CoV-2, accompanied by basement membrane disruption [49]. In other pathophysiologies, such as cerebral ischaemic BBB damage, the host gelatinases MMPs have been shown to mediate the degradation of several TJ proteins, including occludin, claudin-5, and zonula occludens-1 [50–53]. These results indicate that the metalloprotease SspA is the enzyme responsible for occludin and collagen IV degradation under our experimental conditions and is the primary cause of the alterations in BBB permeability occurring during *R. anatipestifer*-associated meningitis.

After bacterial invasion of the BBB, the stimulation of host immune pathways is essential for the progression of meningitis. The inflammatory factors released by brain endothelial cells, microglia, astrocytes, and infiltrating immune cells can increase vascular permeability and destroy the integrity of the CNS [54, 55]. Similar to the pathological changes occurring in other types of bacterial meningitis, *R. anatipestifer*-mediated BBB disruption leads to severe inflammation in the brain, with high expression of a large number of inflammatory genes [56, 57]. In a previous study, *R. anatipestifer* infection was found to result in the upregulation of IL-1β, IL-2, IL-4, IL-17A, IL-17D, IL-17F, TLR3, TLR4, and TGF-β expression in duckling brain tissue [41]. In the present study, we found that SspA induced the upregulation of proinflammatory factors, including IL-1β, IL-6, IL-8, IL-17A, CCL3, CCL4, CCL5, and CX3CL1. In addition, SspA not only induced an excessive response of proinflammatory factors but also caused a strong response to anti-inflammatory cytokines, such as IL-10, in the duckling brain. Moreover, disrupting SspA expression did not result in increased expression of these inflammatory factors. These results indicated that *R. anatipestifer* induced strong dysregulation of the cytokine response, and this effect was associated with SspA expression. Although an inflammatory response may be beneficial as a host defence against bacterial infections, excessive inflammation can exacerbate the symptoms of bacterial meningitis and result in the eruption of a “cytokine storm” [58, 59].

CAMs can mediate the capture, docking, and CNS transmigration of immune cells (e.g., leukocytes) [60] such that infiltrating immune cells mediate further inflammatory responses in the brain [61]. Leukocytes are considered a source of large quantities of proinflammatory cytokines and chemokines in the brain [55]. In this study, we found that SspA dramatically increased the expression of CAMs, such as VCAM-1, SELE, and SELP, both in vivo and in vitro. After *R. anatipestifer* entered the CNS, the expression of integrin β2 (ITGβ2) and its ligand (VCAM-1) was significantly increased in vivo, indicating the accumulation of a very large number of lymphocytes in the duck brain.

In summary, *R. anatipestifer* enters the duck CNS and causes encephalitis, which is characterized by SspA-mediated BBB destruction. In this pathophysiological process, SspA first degrades the BBB component occludin, a TJ protein, and the BM protein collagen IV, resulting in BBB destruction and an increase in BBB permeability. Subsequently, cytokines and CAMs are upregulated to recruit large numbers of immune cells to the brain, and these cells further secrete large amounts of cytokines that further destroy the BBB. The evidence presented above indicates that the paracellular route may be the crucial route by which *R. anatipestifer* crosses the BBB into the CNS. Understanding the mechanisms of SspA-induced BBB disruption could lead to the identification of a potential target for therapeutic intervention in CNS diseases caused or aggravated by BBB damage. The findings of the present study may provide insight for developing new treatment strategies to protect the BBB against *R. anatipestifer*-mediated damage.

## Data Availability

This study includes no data deposited in external repositories. Original data and materials will be available from the corresponding author upon request.
